# Prognostic model based on stem cells and oxidative stress related genes for ovarian cancer

**DOI:** 10.1097/MD.0000000000047461

**Published:** 2026-02-20

**Authors:** Kaiyun Qin, Weilan Liu, Liyun Song, Xingshuang Gao, Yan Jiang, Junmei Zhang, Zhaoping Chu

**Affiliations:** a Department of Gynecology, Hebei General Hospital, Shijiazhuang City, Hebei Province, China; b Physical Examination Center, Hebei General Hospital, Shijiazhuang City, Hebei Province, China; c Department of Obstetrics, Hebei General Hospital, Shijiazhuang City, Hebei Province, China.

**Keywords:** cancer stem cells, ovarian cancer, oxidative stress, prognostic model

## Abstract

Ovarian cancer (OC) is a common gynecological condition. Cancer stem cells (CSCs) are tumor cells with the potential to differentiate and self-renew. The aim of this study was to identify genes relevant to stem cells and oxidative stress (OS) in OC and to construct corresponding prognostic models. OS-related genes were obtained from GenBank. The mRNAsi-OS differentially expressed genes (DEGs) were filtered by overlapping OS-related genes, DEGs associated with mRNAsi, and DEGs in OC. Then, the Absolute Shrinkage and Selection Operator (LASSO) algorithm and univariate Cox regression were adopted to construct an OS-mRNAsi-related prognostic model. Subsequently, we validated the predictive value of the model using both the training and validation sets. The differences in immune infiltration and immunotherapy between the OS-CSC-related high- and low-risk subgroups were further explored. Finally, we analyzed the drug sensitivity between the 2 subgroups. A total of 5 prognostic genes (*PLK2*, *CACNA1C*, *PENK*, *NR0B*1, and *HNF4A*) related to CSC and OS were screened. The area under the curve (AUC) value of the prognostic model in predicting the 3-, 5-, and 7-year survival rate of patients with OC was >0.6, which revealed that the efficiency of the prognostic model was acceptable. The results of CIBERSORT demonstrated noticeable differences in the tumor microenvironment between the OS-CSC-related high- and low-risk subgroups. In addition, the risk score obtained based on OS and mRNAsi can be used to estimate the effectiveness of immunotherapy in patients with OC. Finally, the sensitivity of 5 common drugs (docetaxel, cisplatin, doxorubicin, mitomycin C, and paclitaxel) was evaluated using an OS-CSC-related prognostic model. In conclusion, an OS-CSC-related prognostic model based on 5 genes (*PLK2*, *CACNA1C*, *PENK*, *NR0B*1, and *HNF4A*) was constructed using bioinformatics analysis, which may provide new insights into the treatment and evaluation of OC.

## 1. Introduction

Ovarian cancer (OC) is the deadliest gynecological cancer, affecting more than 300,000 women and causing 152,000 deaths each year.^[1]^ In our country, the mortality rate of OC has shown a rapidly increasing trend, resulting in enormous psychological and economic burdens on patients and society.^[2]^ OC has significant morbidity and mortality partly because it is often diagnosed at an advanced stage.^[3]^ Late diagnosis is a determinant of poor prognosis for OC due to the absence of specific symptoms and reliable biomarkers for early detection.^[4]^ Currently, only a limited number of biomarkers such as cancer antigen 125 (CA125) and human epididymis protein 4 (HE4), are clinically used. However, their specificity is low, and a singular testing strategy is unlikely to achieve satisfactory results.^[5]^ Thus, it is imperative to identify novel diagnostic and prognostic biomarkers and innovative therapeutic targets for OC to improve patient outcomes.

Cancer stem cells (CSCs) are tumor cells with self-renewal and differentiation abilities. Frequent characteristics of CSCs include sustained proliferation, invasion of normal tissue, promotion of angiogenesis, and evasion of the immune system. These characteristics often lead to tumor progression, therapeutic resistance, and recurrence.^[6,7]^ Tumor cells produce more reactive oxygen species (ROS) because of their rapid proliferation and inadequate vascular perfusion.^[8]^ ROS serve as signal-transducing messengers in certain redox-sensitive molecular pathways that are implicated in cell survival, therapeutic resistance, progression, and DNA damage by diffusing through the mitochondrial membrane.^[9]^ Studies have shown that oxidative stress (OS) plays a critical role in regulating stem cell function.^[10,11]^ The effects of OS on stem cells have been demonstrated in various cell types, including hematopoietic stem cells,^[11]^ breast cancer cells,^[12]^ glioma cells,^[13]^ and colorectal cancer cells.^[14]^ Numerous studies have investigated the association between CSCs and OC^[15]^ and the role of OS in OC.^[16]^ However, OC prognosis is influenced by numerous factors, and the molecular mechanisms underlying OC are intricate and multifaceted. Hence, the exploration of polygene-based models may lead to more optimized results. In recent years, increasing evidence has highlighted the critical roles of cancer stemness and oxidative stress in OC progression and therapeutic resistance. However, previous studies have mainly focused on these 2 biological processes independently, and their combined prognostic value in OC remains largely unexplored. In recent years, increasing evidence has highlighted the critical roles of cancer stemness and oxidative stress in OC progression and therapeutic resistance. However, previous studies have mainly focused on these 2 biological processes independently, and their combined prognostic value in OC remains largely unexplored. To our knowledge, this study represents the first systematic integration of the mRNA expression-based stemness index (mRNAsi), oxidative stress–related pathways, and clinical prognosis in OC.** By jointly analyzing mRNAsi-associated genes, oxidative stress–related gene sets, and differentially expressed genes (DEGs) between OC and normal tissues, we identified a subset of characteristic genes with potential biological relevance to both stemness maintenance and oxidative stress regulation. Based on these genes, a novel prognostic model was constructed to provide a more comprehensive and biologically interpretable risk stratification for patients with OC.

This study aims to investigate the prognostic significance and potential molecular regulatory mechanisms of stem cells and OS-related genes in OC. To provide a novel strategy for the prognosis prediction and treatment of OC.

## 2. Materials and methods

### 2.1. Data source

This study was approved by the Ethics Committee of Hebei General Hospital.Clinical and RNA-seq data of 378 patients with OC were extracted from The Cancer Genome Atlas (TCGA) database.^[17]^ The RNA-seq datasets (GSE40595, GSE66957, and GSE53963) were obtained from the Gene Expression Omnibus (GEO) database. The GSE40595 (ovarian stromal tissue, 31 OC samples, and 8 normal control (NC) samples) and GSE66957 datasets (ovarian tissue, 57 OC samples, and 12 NC samples) were used for differential expression analysis. The GSE53963 dataset (ovarian tissue, 174 OC samples) was used as the external validation set. In addition, 3840 OS genes were extracted from the GenBank database (relevant > 2).

### 2.2. The mRNAsi analysis of samples in TCGA database

According to the stem cell expression profile in the Progenitor Cell Biology Consortium (PCBC) database (syn2701943), mRNAsi was calculated using a one-class linear regression (OCLR) algorithm. Then, the transcriptome expression corresponding to the mRNAsi was applied to the TCGA database to determine the mRNAsi of each sample. Subsequently, the median risk score was determined, and patients with OC were stratified into low-mRNAsi and high-mRNAsi groups. Differences in clinical characteristics such as age, tumor stage, and survival status between the 2 groups in the TCGA samples were determined using the chi-square test. The Kaplan–Meier (K–M) curve was used to assess the survival disparity between the 2 mRNAsi groups using Survminer (version: 0.4.9; Vienna, Austria).^[18]^ Additionally, the DEGs between the 2 mRNAsi groups were acquired using DESeq2 (version: 1.34.0; Seattle) (adj. *P* < .05, |log_2_FC| > 1).^[19]^ The mRNA expression-based stemness index (mRNAsi) of each OC sample was calculated using the one-class logistic regression (OCLR) algorithm based on the stem cell expression profiles obtained from the PCBC database. To stratify patients according to stemness characteristics, the median mRNAsi value of all OC samples in the TCGA cohort was used as the cutoff. Samples with mRNAsi values above the median were classified into the high-mRNAsi group, whereas those with mRNAsi values equal to or below the median were assigned to the low-mRNAsi group. This median-based stratification strategy ensured balanced group sizes and has been widely applied in previous prognostic and stemness-related bioinformatics studies.

### 2.3. Analysis of differential genes and enrichment function

The limma package (version: 3.50.1; Seattle) was used to extract DEGs in OC and NC specimens from the GSE40595 and GSE66957 datasets (adj. *P* < .05).^[20]^ Genes with increased or decreased expression among the DEGs obtained from the 2 datasets were intersected. Subsequently, the DEGs in OC were identified by merging the overlapping genes. The mRNAsi-OS-DEGs were filtered by overlapping DEGs associated with mRNAsi, OS-related genes, and DEGs in OC. In addition, GO and KEGG enrichment analyses were performed using the ClusterProfiler package (version: 4.2.2; Buffalo) (adj. *P* < .05).^[21]^

### 2.4. Risk score-based subgroup analysis of OC patients

Firstly, on the basis of survival information of 378 OC samples in the TCGA database and mRNAsi-OS-DEGs, prognostic genes were received by the Absolute Shrinkage and Selection Operator (LASSO) algorithm and univariate Cox regression. The Coxph function in the survival package (version 3.3.1; Vienna, Austria) was used for univariate Cox regression analysis (HR = 1, *P* < .05).^[22]^ The LASSO algorithm was implemented using the glmnet package (version 4.1.2; Vienna, Austria).^[23]^ Prognostic genes were analyzed by the Spearman correlation analysis. Then, the coefficients of the genes obtained from the LASSO analysis and their expression values were calculated using the following formula:


$$\begin{array}{l} risk\;score=\sum_{i=1}^{n}\beta i*xi \end{array}$$

After obtaining the median risk score, 378 patients with OC were stratified into OS-CSC-related low- and high-risk groups. Then, dimensionality reduction analysis was performed via Principal component analysis and t-distributed stochastic neighbor embedding. For survival analysis of the 2 risk score groups, K–M curves were plotted using Survminer (version 0.4.9). To further assess the effectiveness of the prognostic model, the survival ROC package (version 1.0.3) was used to paint the ROC curve with 3, 5, and 7 years as the survival time node.^[24]^ The GSE53963 dataset was used as an external validation dataset to validate the prognostic model.

### 2.5. Clinical nomogram model

Independent risk factors associated with the prognosis of OC were acquired using COX regression (univariate and multivariate) analyses according to the clinical data of patients with OC and the risk score. Subsequently, a nomogram was created to predict the survival rates of OC patients at 3, 5, and 7 years based on independent factors. The predictive effect of the nomogram was evaluated using DCA, ROC, and calibration curves.

### 2.6. Functional annotation of OC specimens

Functional enrichment analysis of the 2 risk score groups was performed using GSVA software (https://github.com/rcastelo/GSVA). Reference gene sets were obtained from the MSigDB database.

### 2.7. Analysis of tumor microenvironment and immune infiltration

The estimate package (version 1.0.13; Vienna, Austria) was used to calculate tumor purity, immune score, stromal score, and ESTIMATE score for patients with OC.^[25]^ The ratio of 22 immune cell species in each OC sample in TCGA database was analyzed using the CIBERSORT algorithm. Differential immune cells in the 2 risk score subgroups were selected using the Wilcoxon test. The association between prognostic genes and differential immune cells was determined using the Spearman correlation analysis.

### 2.8. Analysis of immunotherapy response

The differential immune checkpoint genes in the 2 risk groups and their associations with risk scores were examined. Tumor Immune Dysfunction and Exclusion (TIDE) scores were calculated using the TIDE network. We also analyzed the relationship between the risk and the TIDE scores. Subsequently, an association analysis between the risk score and cytolytic activity score (CYT), granzyme A (GZMA), and perforin 1 (PRF1) was performed.

### 2.9. Sensitivity analysis of chemotherapy drugs

The half-maximal inhibitory concentrations (IC50) of 138 drugs were calculated using the pRRophetic package (version 0.5; Chicago).^[26]^ We then analyzed the IC50 differences between the 2 subgroups of several chemotherapy drugs that are frequently used in OC. The expression levels of target genes predicted by the DrugBank database of differential drugs were analyzed in the 2 subgroups (https://www.drugbank.ca).

### 2.10. Quantitative real-time polymerase chain reaction (qRT-PCR)

Fifty milligram of tissue was taken from each sample, TRIzol reagent was added to fully lyse cells to extract total RNA, and 1 ul was taken for concentration detection by Nano drop. Reverse transcription into cDNA was performed using the SureScript-First-strand-cDNA-synthesis kit (Servare, Murray). Amplification was performed in a 10 ml system containing 2 × Universal Blue SYBR Green qRT-PCR Master Mix (Servare). In 2^−ΔΔCT^ method to calculate the relative expression of the gene.

### 2.11. Statistical analysis

All open databases and R software (version 4.1.0; Vienna, Austria) were used for the analysis and visualization in this study. A heat map was created using the pheatmap package (version 1.0.12; Vienna, Austria).^[27]^ A significant difference was considered when *P* < .05.

## 3. Results

### 3.1. Identification of mRNAsi-related DEGs in OC

**Figure 1.**
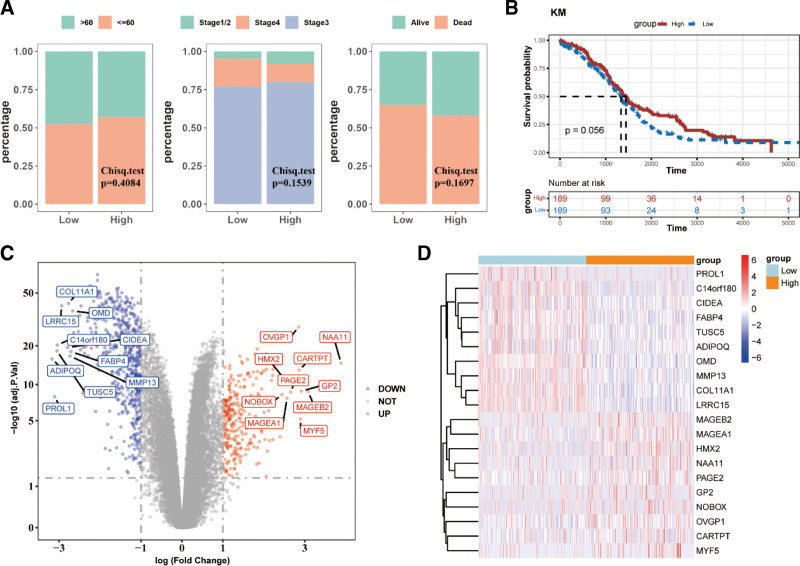
Identification of the mRNAsi-related DEGs in ovarian cancer (OC). (A) Clinical traits between the 2 mRNAsi groups were not significantly different according to the chi-square test; (B) Kaplan–Meier curves showing the survival probability between the 2 mRNAsi groups; (C) Volcano plots showing DEGs between the 2 mRNAsi groups; (D) Heat map showing changes in the expression levels of the top 20 genes. DEGs = differentially expressed genes.

mRNAsi analysis was performed to quantify the probability of tumor cells developing into cancer cells in OC. The chi-square test showed that the differences observed in clinical traits (age, tumor stage, and survival status) between the 2 mRNAsi groups were not significant (Fig. 1A). Similarly, the K–M curve showed that the survival probability of the high-mRNAsi group was slightly different from that of the other groups (Fig. 1B). A total of 829 DEGs were identified between the 2 mRNAsi groups, with 269 genes showing increased expression and 560 showing decreased expression in the high-mRNAsi group (Fig. 1C). The top 20 genes whose expression levels changed were visualized on a heatmap (Fig. 1D).

### 3.2. Identification of mRNAsi-OS-DEGs and enriched functions and pathways

**Figure 2.**
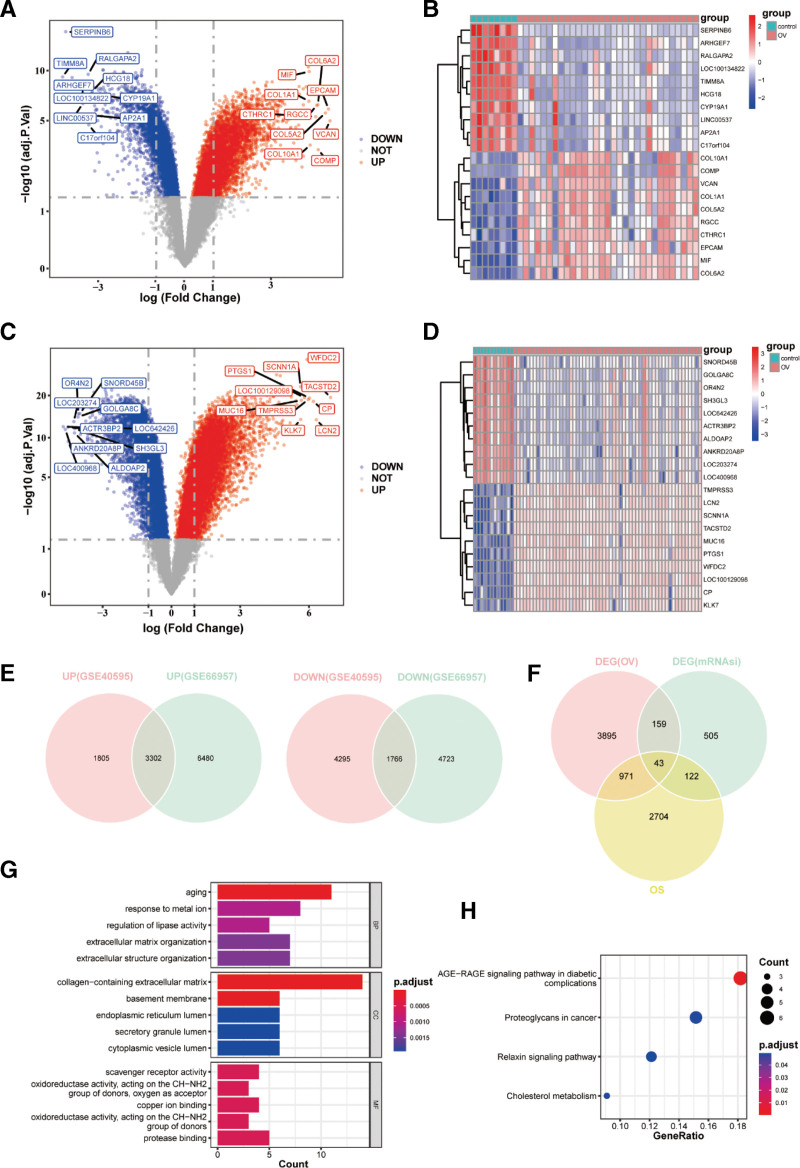
Identification of mRNAsi-OS-DEGs and enriched functions and pathways. (A) Volcano plots show DEGs in GSE40595 dataset; (B) Heat map shows the DEGs were obtained in GSE40595 dataset; (C) Volcano plots show DEGs in GSE66957 dataset; (D) Heat map shows the DEGs were obtained in GSE66957 dataset; (E) the intersection of DEGs in 2 datasets; (F) mRNAsi-OS-DEGs are filtered by overlapping OS-related genes, DEGs associated with mRNAsi and DEGs in ovarian cancer (OC); (G) the result of GO enrichment analysis; (H) the result of KEGG enrichment analysis. DEGs = differentially expressed genes, GO = gene ontology, KEGG = Kyoto Encyclopedia of Genes and Genomes, OS = oxidative stress.

A total of 11,168 DEGs were obtained from the GSE40595 dataset, of which 5107 genes exhibited high expression and 6061 genes exhibited low expression in the OC samples (Fig. 2A). The top 20 genes are shown in Figure 2B. In addition, 16,271 DEGs were identified in the GSE66957 dataset. The OC samples showed upregulation of 6489 genes and downregulation of 9782 genes (Fig. 2C); the top 20 genes are shown in Figure 2D. A total of 5068 DEGs in the OC samples were identified by taking the intersection of the DEGs in the 2 datasets (Fig. 2E). Subsequently, 43 mRNAsi-OS-DEGs were filtered by overlapping 3840 OS-related genes, 829 DEGs associated with mRNAsi, and 5068 DEGs associated with OC (Fig. 2F). To investigate the potential functions and pathways of mRNAsi-OS-DEGs, GO and KEGG enrichment analyses were performed. According to the GO results, “aging,” “response to metal ions,” “endoplasmic reticulum lumen,” and ‘scavenger receptor activity’ were the main enriched mRNAsi-OS-DEGs (Fig. 2G). Moreover, in KEGG terms, “proteoglycans in cancer,” “cholesterol metabolism,” and “relaxin signaling pathway” were relevant to mRNAsi-OS-DEGs (Fig. 2H).

### 3.3. The OS-CRC-related prognostic model based on mRNAsi-OS-DEGs

**Figure 3.**
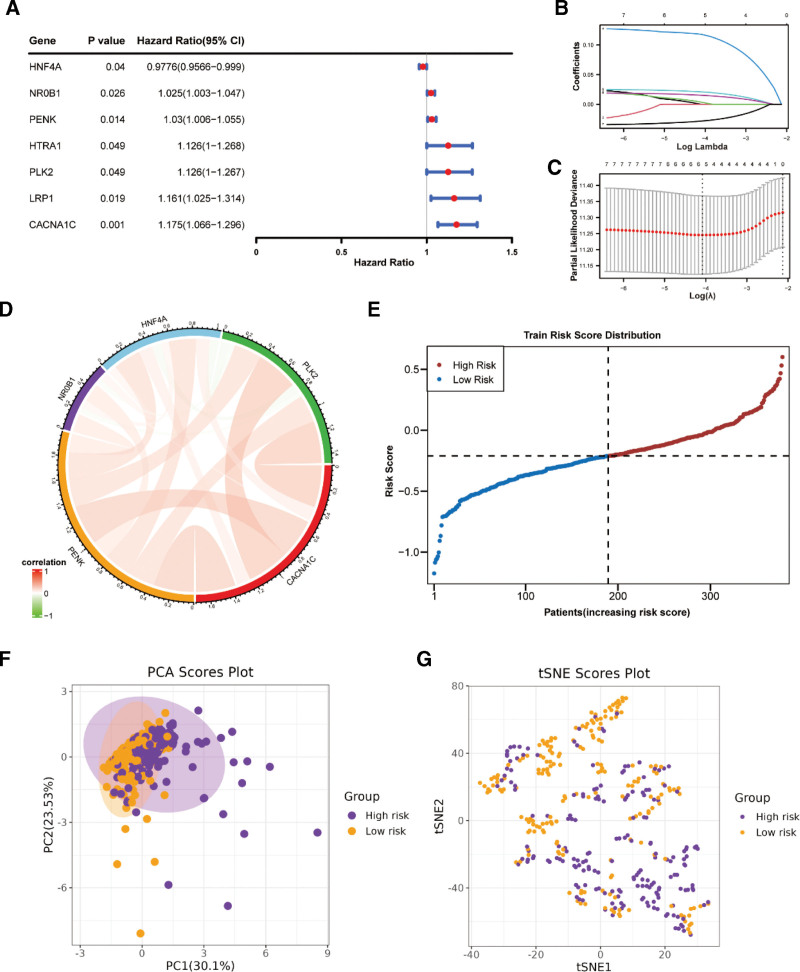
The OS-CSC-related prognostic model based on mRNAsi-OS-differentially expressed genes (DEGs). (A) Univariate Cox regression selected out the genes; (B and C) LASSO algorithm screen out 5 prognostic genes (*PLK2*, *CACNA1C*, *PENK*, *NR0B*1, and *HNF4A*); (D) Correlation analysis results of prognostic genes; (E) Distribution of OS-CSC-related low- and high-risk groups based on risk score; (F) PCA analysis distinguish between 2 risk groups; (G) The tSNE analysis distinguished between 2 risk groups. CSC = cancer stem cells, OS = oxidative stress, PCA = principal component analysis, tSNE = t-distributed stochastic neighbor embedding.

**Figure 4.**
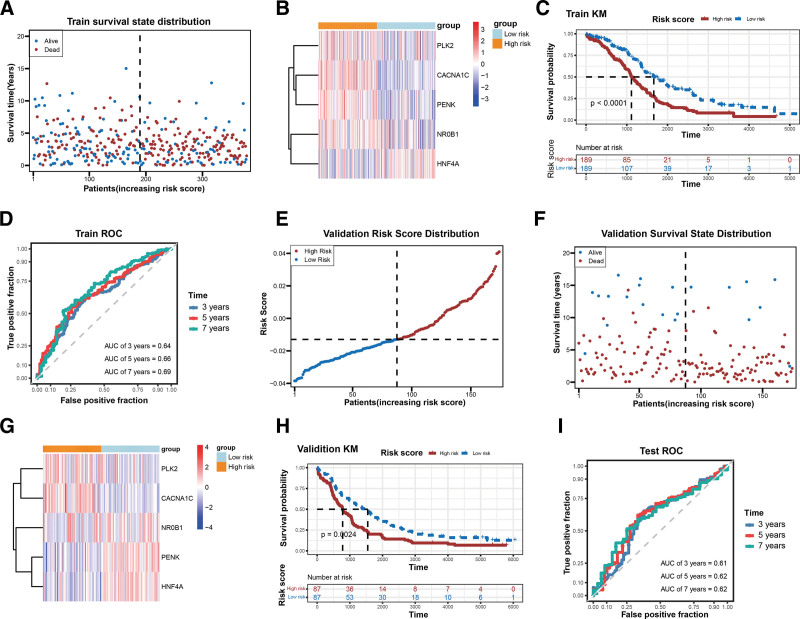
Association of prognostic genes with survival; (A) the differences of survival state distribution and prognostic genes expression; (B) the expression of prognostic genes in 2 risk groups; (C) Kaplan–Meier curve show survival probability between 2 risk groups; (D) the AUC value of the prognostic model for predicting 3-, 5-, and 7-year survival in OC patients; (E) distribution of patients of 2 risk groups in validation dataset; (F) the survival state distribution of 2 risk groups in validation dataset; (G) the expression of prognostic genes in 2 risk groups in validation dataset; (H) Kaplan–Meier curve show survival probability between 2 risk groups in validation dataset; (I) the AUC value of the prognostic model for predicting the 3-, 5-, and 7-year survival in OC patients in validation dataset. AUC = area under the curve, OC = ovarian cancer.

In total, 7 genes (*PLK2*, *CACNA1C*, *PENK*, *NR0B1*, *HNF4A*, *HTRA1*, and *LRP1*) were selected using univariate Cox regression (Fig. 3A). Five prognostic genes (*PLK2*, *CACNA1C*, *PENK*, *NR0B*1, and *HNF4A*) were screened using the LASSO algorithm (Fig. 3B and C). Table 1 shows the coefficients of 5 prognostic genes. Correlation analysis results for the prognostic genes are shown in Figure 3D. Next, the patients were stratified into OS-CRC-related low- and high-risk groups based on the risk score (Fig. 3E). Principal component analysis and t-distributed stochastic neighbor embedding analyses could significantly distinguish between the 2 risk groups, as shown in Figure 3F and G. Figure 4A and B presents the differences in survival state distribution and prognostic gene expression. Four prognostic genes (*PLK2*, *CACNA1C*, *PENK*, and *NR0B1*) were highly expressed and only *HNF4A* was expressed at low levels in the high-risk group (Fig. 4B). The K–M curve indicated that the high-risk group was associated with a lower survival probability (Fig. 4C). The area under the curve values (0.64, 0.66, and 0.69) of the prognostic model in predicting the 3-, 5-, and 7-year survival rate of patients with OC revealed that the efficiency of the prognostic model was acceptable (Fig. 4D). The same conclusion was obtained for the external validation set GSE53963 of the prognostic model (Fig. 4E–I).

**Table 1:** Coefficients of the prognostic genes.

Lasso gene	Coef
PLK2	0.003369962
CACNA1C	0.117706151
PENK	0.020892761
NR0B1	0.015123756
HNF4A	−0.028263238

### 3.4. Clinical nomogram model

**Figure 5.**
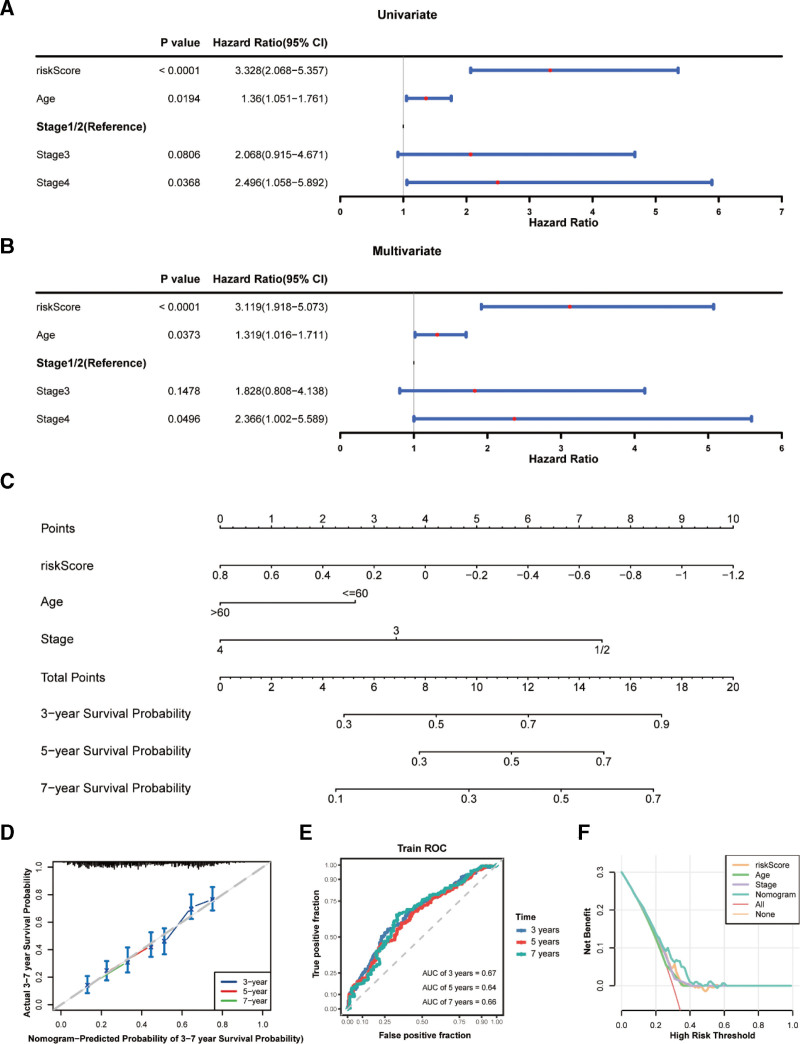
Clinical nomogram is constructed to combine with clinical factors. (A and B) univariate analysis; (A) and multivariate analysis; (B) confirm independent risk elements; (C) clinical nomogram is established base on the risk factors. (D–F) The validity of the model is verified by the DCA curve (D), ROC curve (E), and calibration curve (F).

Based on the risk score and clinical data of patients with OC, a clinical nomogram was constructed and combined with clinical factors. The stage, risk score, and age were independent risk factors for OC patients (Fig. 5A and B). These independent risk factors were used to establish a nomogram to forecast survival probabilities of OC patients at 3, 5, and 7 years (Fig. 5C). In addition, the DCA, ROC, and calibration curves of the nomogram indicated that it could be regarded as an effective model (Fig. 5D–F).

### 3.5. Prognostic model-based study of the molecular mechanisms of OC

**Figure 6.**
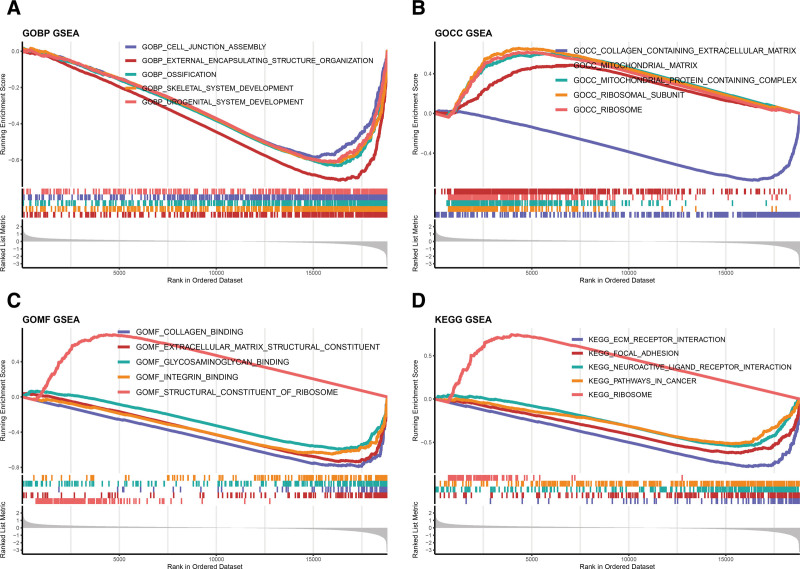
Prognostic model-based study of the molecular mechanisms of OC. (A–C) The result of GO analysis includes molecular function (A), biological process (B), cell component (C). (D) The result of KEGG analysis. GO = gene ontology.

Gene Ontology (GO) and Kyoto Encyclopedia of Genes and Genomes (KEGG) analyses were performed to explore the potential functions and pathways of the 2 risk subgroups. As for the GO results, the samples in low-risk group were enriched in “cell junction assembly,” “integrin binding,” “external encapsulating structure organization,” “extracellular matrix structural constituent,” and functions related to the ribosome and mitochondria were enriched in high-risk group (Fig. 6A–C). Moreover, in KEGG terms, the high-risk group was interrelated to “ribosome” and the low-risk group was associated with “pathways in cancer,” “ECM receptor interaction,” and “focal adhesion” (Fig. 6D).

### 3.6. The high-risk group was accompanied by lower tumor purity

**Figure 7.**
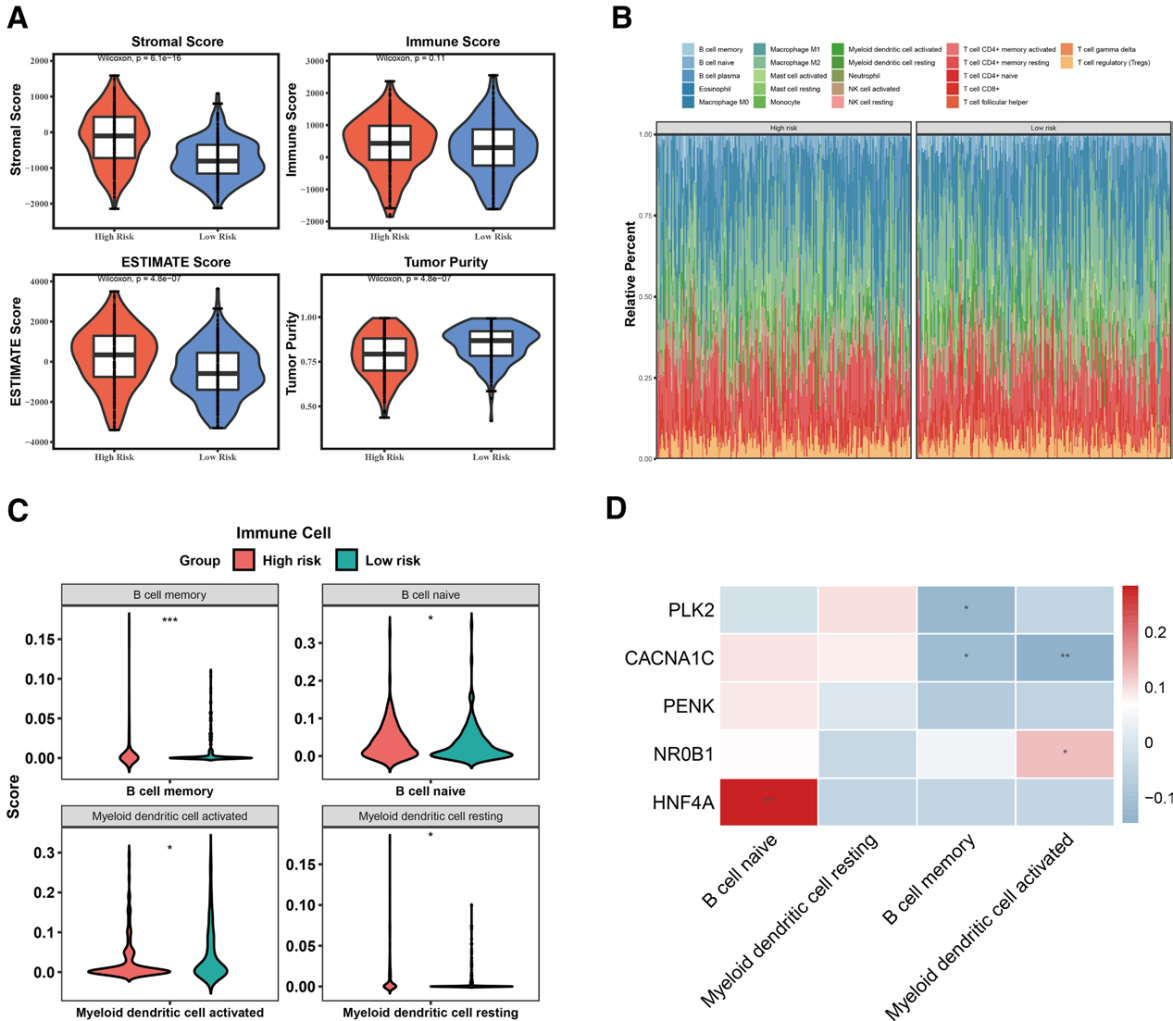
The relationship of prognosis genes and the immune microenvironment in OC. (A) Wilcoxon test evaluates immune score, tumor purity, ESTIMATE score, and stromal score between 2 risk score groups; (B) the result of CIBERSORT analysis; (C) differential immune cells are identified between 2 risk score groups; (D) Spearman correlation analysis shows the correlation between prognostic genes and differential immune cells. OC = ovarian cancer.

Because the prognosis of tumors is intimately interrelated with the immune microenvironment, we also investigated the relationship between prognostic genes and the immune microenvironment in OC. The Wilcoxon test revealed that tumor purity, ESTIMATE score, and stromal score were significantly different between the 2 risk score groups, except for the immune score (Fig. 7A). The results of CIBERSORT analysis is displayed in Figure 7B. Four different immune cells (resting myeloid dendritic cells, activated myeloid dendritic cells, naïve B cells, and memory B cells) were identified between the 2 risk score groups using the Wilcoxon test (Fig. 7C). Additionally, *HNF4A* and *NR0B1* were positively associated with naïve B cells and activated myeloid dendritic cells, respectively. *PLK2* and *CACNA1C* were negatively correlated with memory B cells, and *CACNA1C* was negatively correlated with activated myeloid dendritic cells based on the Spearman correlation analysis (Fig. 7D).

### 3.7. Predictive value of risk scores for immunotherapy

**Figure 8.**
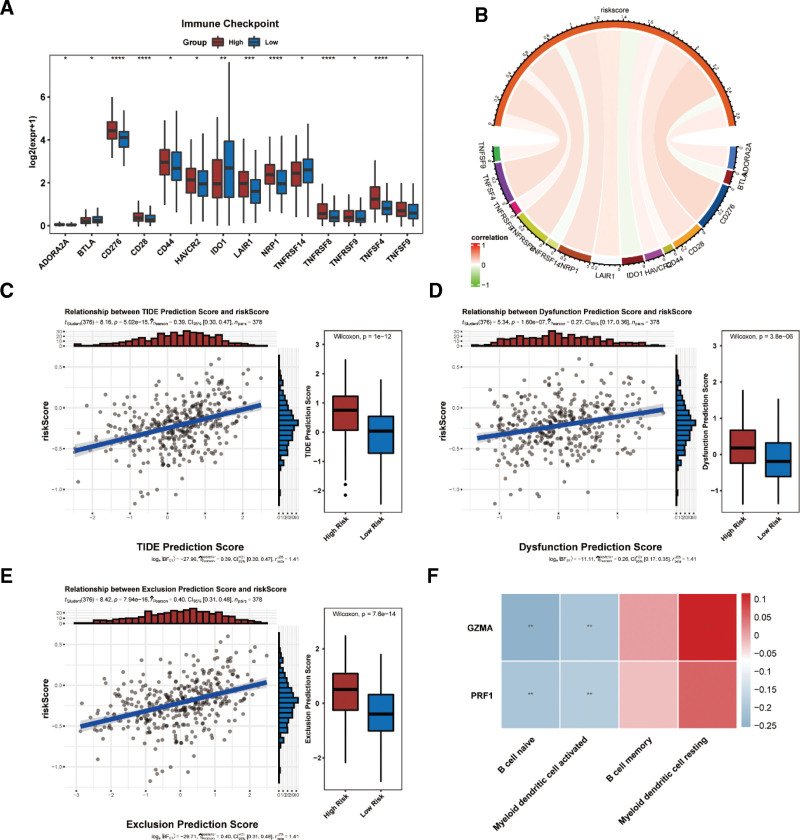
Predictive value of risk scores for immunotherapy. (A) The expression differences of 14 immune checkpoint genes are noticeable between 2 risk score groups; (B) correlation analysis between differential immune checkpoint genes and the risk score. (C–E) The relationship between the predicted score of Tumor Immune Dysfunction and Exclusion (TIDE) (C), dysfunction (D), and exclusion (E) and the risk score; (F) correlation analysis between differential immune cells and GZMA, PRF1.

Owing to the effectiveness of immunotherapy in cancer treatment, the association between the risk score and immunotherapy was investigated. The expression differences of 14 immune checkpoint genes (*ADORA2A, BTLA, CD276, CD28, CD44, HAVCR2, IDO1, LAIR1, NRP1, TNFRSF14, TNFRSF8, TNFRSF9, TNFSF4*, and *TNFSF9*) were noticeable between the 2 risk score groups (Fig. 8A). Additionally, most of the differential immune checkpoint genes were positively associated with the risk score (Fig. 8B). The predicted scores of TIDE, dysfunction, and exclusion were positively associated with the risk score, and significant differences were present in the 2 subgroups (Fig. 8C–E). However, there was no significant correlation between the risk score and the CYT, GZMA, or PRF1 scores. Moreover, the correlation analysis revealed that activated myeloid dendritic cells and naïve B cells were negatively associated with GZMA and PRF1 (Fig. 8F).

### 3.8. Drug sensitivity analysis and target gene prediction

**Figure 9.**
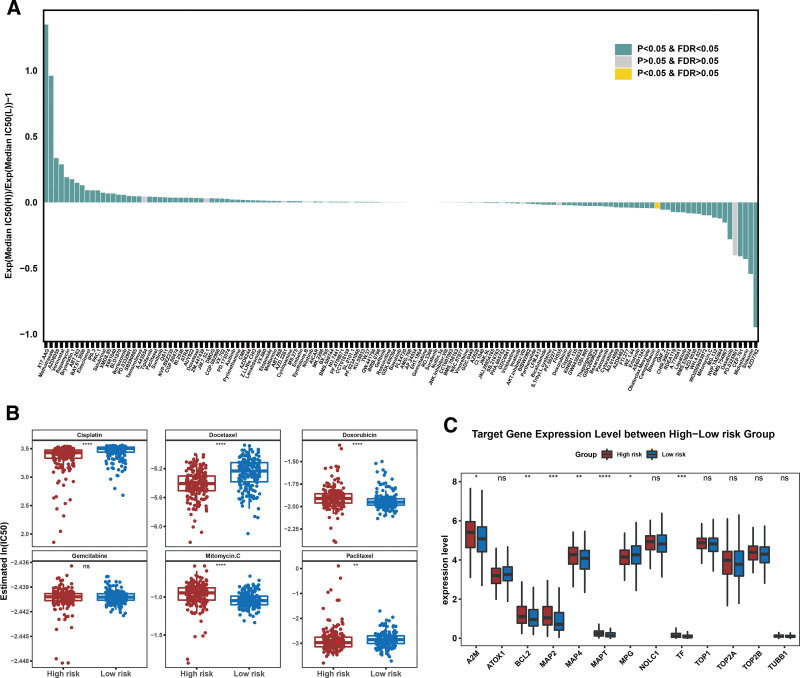
Drug sensitivity analysis and target gene prediction. (A) The differential is screened for IC50 in 117 drugs between 2 risk score groups; (B) difference analysis of 6 commonly used chemotherapy drugs in ovarian cancer (OC) between 2 risk score groups; (C) the expression of target genes predicted by DrugBank database was different between the 2 risk score groups.

Drug sensitivity analysis was conducted to identify effective therapeutic drugs. In summary, 117 drugs were screened for their IC50 values, which differed between the 2 subgroups (Fig. 9A). Six chemotherapeutic drugs (paclitaxel, cisplatin, gemcitabine, docetaxel, doxorubicin, and mitomycin C) commonly used in OC were screened for further analysis. As shown in Figure 9B, 5 drugs (cisplatin, docetaxel, doxorubicin, mitomycin C, and paclitaxel) showed significant differences between the 2 risk score groups, except for gemcitabine. In addition, 13 target genes were predicted using the DrugBank database, of which the expression of 7 (A2M, BCL2, MAP2, MAP4, MAPT, MPG, and TF) differed between the 2 subgroups (Fig. 9C).

### 3.9. Expression of the prognostic genes

**Figure 10.**
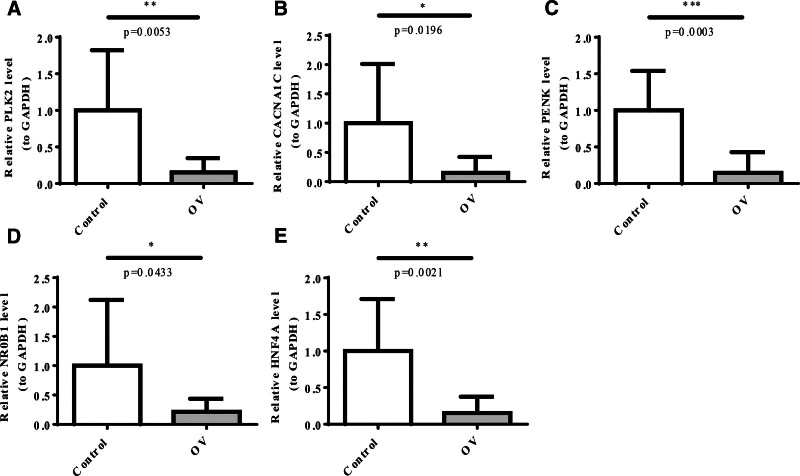
Expression of the prognostic genes. (A–E) qRT-PCR analysis of expression level of *PLK2* (A), *CACNA1C* (B), *PENK* (C), *NR0B1* (D), and *HNF4A* (E) between ovarian cancer (OC) tissues and normal adjacent tissues.

To investigate the expression of 5 prognostic genes (*PLK2*, *CACNA1C*, *PENK*, *NR0B*1, and *HNF4A*), we examined their expression in OC tissues by qRT-PCR and compared them with that in normal adjacent tissues. Our data showed that compared with normal adjacent tissues, the expression of all 5 genes was significantly decreased in OC (Fig. 10A–E).

## 4. Discussion

The major innovation of the present study lies in the systematic integration of tumor stemness and oxidative stress in the prognostic evaluation of OC. Unlike previous studies that primarily investigated stemness or oxidative stress in isolation, we combined the mRNA expression-based stemness index (mRNAsi) with oxidative stress–related pathways to identify genes that simultaneously reflect these 2 critical biological characteristics. This integrative strategy enabled the screening of prognostic genes with clearer biological significance and improved the interpretability of the prognostic model. OC is one of the deadliest gynecologic malignancies in the world.^[28]^ In the early stages of the disease, OC lacks obvious clinical manifestations, resulting in the majority of patients being diagnosed at advanced stages and ultimately succumbing to the disease due to recurrence and metastasis.^[28]^ Therefore, reliable prediction of patient prognosis using biomarkers is highly valuable. In this study, we used the LASSO-Cox algorithm to identify 5 genes (*PLK2, CACNA1C, PENK, NR0B1* and *HNF4A*) associated with stemness and oxidative stress in OC cells and constructed prognostic models based on these genes. Previous studies have also developed prognostic models for OC based on genes associated with hypoxia or the tumor microenvironment.^[29,30]^ Our study used a novel approach by not only screening for DEGs between OC and normal tissues, but also according to the stem cell expression profile in the PCBC database (syn2701943). The mRNAsi calculated by the OCLR algorithm was applied to the TCGA database to divide the patients into a low-mRNAsi group and a high-mRNAsi group. The prognostic model demonstrated excellent performance in the external validation and successfully established a clinical nomogram.

Chakrabarty and Chandel review of the current understanding of ROS and oxidative stress-regulated stem cells enhanced our understanding of cancer.^[31]^ Tuy et al conducted a review on the effects of ROS on CSCs.^[32]^ They suggest that ROS are produced by various cellular processes, act on growth-related genes and pathways, and contribute to uncontrolled proliferation, which is a characteristic of malignancy. Throughout tumorigenesis, cancer cells must withstand the various stages of different stages.^[33]^ ROS plays a crucial role in hypoxia.^[34,35]^ Stem cells and their tissue niches are critical for tissue repair and homeostasis. High oxygen tension levels may render stem cells susceptible to DNA damage induced by oxidative stress.^[36]^ Hypoxia plays an important role in stem cell self-renewal.^[37]^ Studies on the redox characterization of CSCs and ROS-related processes are ongoing. The objective of our study was to identify DEGs associated with stemness and oxidative stress in OC cells and subsequently establish prognostic models.

The significance of prognostic genes in tumors and OC has also been reported in other studies. *PLK2* plays a role in tumorigenesis; it is considered as tumor suppressor.^[38]^ For instance, *PLK2* has been shown to exert antiproliferative and proapoptotic effects in cervical cancer.^[39]^ However, in colorectal carcinomas, *PLK2* promotes tumor growth and inhibits apoptosis.^[40]^ The role of *PLK2* in tumorigenesis remains unclear. The expression of *PLK2* was upregulated in the high-risk group. Previous studies have shown that downregulation of *PLK2* is associated with the occurrence and development of ovarian tumors as well as their resistance to treatment.^[41,42]^ *CACNA1C* is a voltage-dependent calcium ion transmembrane channel that exerts regulatory effects on the development and progression of multiple tumors. Chang et al reported that the expression of *CACNA1C* in OC tissues was significantly lower than that in normal tissues. Thus, CACNA1C may be a prognostic predictor of OS in OC.^[43]^ However, the role of *PENK* and *NROB1* in OC remains unclear. PENK inhibits osteosarcoma cell migration.^[44]^ Furthermore, the identification of aberrant methylation patterns in *PENK* shows promise as a potential biomarker for breast cancer screening.^[45]^ Robust *NROB1(DAX-1*) expression in operable node-negative breast cancer is associated with a favorable prognosis. One studies also proposed that *HNF4α* was positive in the ascites of ovarian mucinous adenocarcinoma and proved to be a useful marker for the diagnosis of ovarian mucinous tumors.^[46]^

The impact of the tumor microenvironment on cancer cells has recently garnered increasing attention. This study focused on the relationship between prognostic genes and the immune microenvironment in patients with OC. The CIBERSORT algorithm was used to analyze immune infiltration. Resting myeloid dendritic cells, activated myeloid dendritic cells, naïve B cells, and memory B cells were discrepant between the 2 risk score groups. Previous studies have demonstrated the pivotal role of B cell infiltration in tumor immunotherapy.^[47]^ Wei et al observed the presence of a subset of interleukin-10(+) B cells in the ascites of patients has been observed, which is associated with the naïve B cell phenotype, IgM, or class-switched memory B cell phenotypes.^[48]^ Other studies have suggested that chemotherapy reduces the total number of B lymphocytes, especially in specific subsets such as naïve B cells and memory B cells.^[49]^ Correlation analysis showed that *HNF4A* and *NR0B1* were positively associated with naïve B cells and activated myeloid dendritic cells, respectively. *PLK2* and *CACNA1C* were negatively correlated with memory B cells, and *CACNA1C* was negatively correlated with activated myeloid dendritic cells. These findings suggest that CSCs and oxidative stress may affect the tumor microenvironment and that prognostic genes may lead to variations in the tumor microenvironment.

Recently, there has been a surge in research investigating the potential therapeutic applications of immunotherapies and checkpoint inhibitors.^[50]^ Differential immune checkpoint genes (*ADORA2A*, *BTLA*, *CD276*, *CD28*, *CD44*, *HAVCR2*, *IDO1*, *LAIR-1*, *NRP1*, *TNFRSF14*, *TNFRSF8*, *TNFRSF9*, *TNFSF4*, and *TNFSF9*) were screened in the 2 risk groups in our study. Janina et al conducted a study which found that the level of *BTLA* in patients with OC was higher than that in normal individuals, and that *BTLA* was associated with shorter survival.^[51]^ *CD276* is an immune checkpoint molecule in the epithelial-mesenchymal transition (EMT) pathway that plays a critical role in the proliferation, invasion, and migration of malignant tumors.^[52]^ CD44 is a cell surface protein that plays an important role in CSC function and is closely related to the metastatic progression and chemotherapy resistance of ovarian tumors. CD44, a cell surface protein, plays a pivotal role in the functionality of CSCs and is closely associated with metastatic progression and chemotherapy resistance in OC.^[53]^ IDO1 expression can increase the frequency of PD-1CD8 tumor-infiltrating T cells and induce PD-1 expression in T cells in OC.^[54]^ LAIR-1 suppresses OC cell.^[55]^ Thus, NRP1 may be associated with drug resistance in OC.^[56]^ The relationship between other immune checkpoint genes and OC requires further study; however, exploring this avenue could provide a novel direction for future research.

CSCs are commonly resistant to chemotherapy and radiotherapy, and the administration of chemotherapeutic agents or radiation can either directly or indirectly induce oxidative stress. Drug resistance and disease recurrence are the major challenges in cancer treatment.^[57]^ Therefore, we performed drug sensitivity analysis. Five drugs (cisplatin, docetaxel, doxorubicin, mitomycin C, and paclitaxel) and 7 target genes (*A2M*, *BCL2*, *MAP2*, *MAP4*, *MAPT*, *MPG*, and *TF*) differed between the 2 risk groups. Notably, an apparent inconsistency was observed between the expression patterns of the 5 prognostic genes validated by qRT-PCR and their expression trends derived from the TCGA-based RNA-seq data. Several factors may contribute to this discrepancy. First, the TCGA database consists of large-scale, multi-center RNA-sequencing data, which reflect averaged gene expression across heterogeneous tumor samples, whereas qRT-PCR validation was performed on a relatively small number of locally collected specimens. Differences in tumor stage, histological subtype, and treatment history may therefore introduce sample heterogeneity. Second, technical differences between high-throughput RNA sequencing and qRT-PCR platforms may also affect gene expression quantification. RNA-seq provides global transcriptome profiling, while qRT-PCR is highly sensitive to primer design, RNA quality, and normalization strategies. In addition, bulk RNA-seq data represent the combined expression of tumor cells and stromal or immune components, whereas qRT-PCR measurements may be influenced by tissue sampling regions and tumor purity.

Several limitations of the present study should be acknowledged. First, this study was retrospective in nature and primarily based on publicly available datasets, which may introduce inherent selection bias. Second, although multiple datasets from TCGA and GEO were integrated to improve robustness, potential batch effects and platform heterogeneity could not be completely eliminated. Third, the biological functions and molecular mechanisms of the identified prognostic genes were not experimentally validated, and further in vitro and in vivo studies are required to elucidate their roles in OC progression, stemness maintenance, and oxidative stress regulation. In addition, the number of clinical samples used for qRT-PCR validation was relatively small, which may limit the generalizability of the expression results. Moreover, due to the lack of long-term survival follow-up data for the clinical specimens, the prognostic value of these genes could not be further validated at the clinical outcome level. Therefore, future prospective studies with larger sample sizes, functional experiments, and complete follow-up information are needed to confirm and extend our findings.

## 5. Conclusion

The present study identified 5 genes (*PLK2, CACNA1C, PENK, NR0B1*, and *HNF4A*) that showed differential expression through bioinformatic analysis, and a prognostic model was established. We also investigated the relationship between prognostic genes and the immune microenvironment, immunotherapy, drug susceptibility analysis, and target gene prediction. The findings in this study provide new insights for the treatment and evaluation of OC.

## Author contributions

**Conceptualization:** Kaiyun Qin, Weilan Liu, Liyun Song, Xingshuang Gao, Yan Jiang, Junmei Zhang, Zhaoping Chu.

**Data curation:** Kaiyun Qin, Weilan Liu, Yan Jiang, Zhaoping Chu.

**Formal analysis:** Kaiyun Qin, Weilan Liu, Liyun Song, Xingshuang Gao, Yan Jiang, Zhaoping Chu.

**Funding acquisition:** Kaiyun Qin, Liyun Song, Zhaoping Chu.

**Investigation:** Kaiyun Qin, Zhaoping Chu.

**Writing – original draft:** Kaiyun Qin, Junmei Zhang, Zhaoping Chu.

**Writing – review & editing:** Kaiyun Qin, Xingshuang Gao, Junmei Zhang, Zhaoping Chu.
